# Endemic fluoroquinolone-resistant *Salmonella* *enterica* serovar Kentucky ST198 in northern India

**DOI:** 10.1099/mgen.0.000275

**Published:** 2019-06-05

**Authors:** Jaspreet Mahindroo, Duy Pham Thanh, To Nguyen Thi Nguyen, Balvinder Mohan, Siddhartha Thakur, Stephen Baker, Neelam Taneja

**Affiliations:** 1 Post Graduate Institute of Medical Education and Research, Chandigarh, India; 2 Oxford University Clinical Research Unit, Wellcome Trust Major Overseas Programme, Ho Chi Minh City, Vietnam; 3 College of Veterinary Medicine, North Carolina State University, Raleigh, NC, USA; 4 Department of Medicine, University of Cambridge, Cambridge, UK

**Keywords:** non-typhoidal *Salmonella*, fluoroquinolone, antimicrobial resistance, whole-genome sequencing, India

## Abstract

*
Salmonella* *e* *nterica* serovar Kentucky is an emergent human pathogen. Human infection with ciprofloxacin-resistant *S*. *enterica* Kentucky ST198 has been reported in Europe and North America as a consequence of travel to Asia/the Middle East. This is, to the best of our knowledge, the first study reporting the identification of this epidemic clone in India and South Asia.

## Data Summary

Raw Illumina reads of 23 *Salmonella enterica* serovar Kentucky ST198 isolates in this study have been deposited in the European Nucleotide Archive. Additionally, 103 available genomes of *S. enterica* Kentucky ST198 from England (*n*=63), Denmark (*n*=16), the USA (*n*=13), Ireland (*n*=9) and Kuwait (*n*=2) were also included in the analysis. The accession numbers, project numbers and their corresponding metadata are listed in Table S1 (available with the online version of this article).

**Impact Statement**

Non-typhoidal *Salmonella* (NTS) infections are common in India, but due to the lack of a surveillance system, hardly any data are available. *S* *almonella* *e* *nterica* serovar Kentucky is a growing cause of human NTS infections, due to international dissemination of a single sequence type (ST198) exhibiting resistance to ciprofloxacin (Cip^R^). There have been multiple reports of human ST198 cases in Europe and North America; travel to the Middle East or Asia is commonly associated with these infections. We conducted sustained surveillance for NTS organisms associated with human diarrhoeal disease across a large geographical area in North India. Concurrently, we conducted cross-sectional sampling of meat products and farm animals (sheep, goats, pigs and chickens). We used whole-genome sequencing (WGS) to characterize NTS and found the presence of the epidemic Cip^R^ *S*. *enterica* serovar Kentucky clone ST198 in humans, poultry and goats. This is, to the best of our knowledge, the first study reporting the identification of this epidemic clone in India and South Asia. It also highlights the role that animals and humans play in the circulation of emerging antimicrobial-resistant enteric pathogens, and shows how WGS data is vital for integrating international surveillance systems.

## Introduction

Non-typhoidal *Salmonella* (NTS) are a leading cause of gastroenteritis globally, with the World Health Organization (WHO) recognizing the organisms as one of the four major causes of diarrhoeal illnesses [1, 2]. NTS organisms circulate in animal reservoirs and generally induce a self-limiting gastroenteritis in humans after the ingestion of contaminated food and water. The most common serovars associated with human disease are *Salmonella enterica* serovar Enteritidis and *S*. *enterica* serovar Typhimurium, but other serovars appear to be becoming more visible on the global NTS landscape [3]. One such example is *S. enterica* serovar Kentucky, which was first described in poultry populations in 1937 [4]. After its discovery, *S*. *enterica* Kentucky became increasingly distributed throughout agriculture systems but rarely caused human NTS infections in comparison to other serovars. However, *S*. *enterica* Kentucky has been a growing cause of human NTS infections since 2005, which has been largely associated with the international dissemination of a single sequence type (ST198) exhibiting resistance to ciprofloxacin (Cip^R^). There have been multiple reports of human ST198 cases in Europe and North America; travel to the Middle East or Asia is commonly associated with these infections [5–7].

India is a vast country with the second largest population in the world. Conditions like high population density, overcrowding, poor sanitation, low socio-economic status and malnourishment in children make India a key location for the likely emergence and international transmission of diarrhoeal pathogens. However, there are limited prospective surveillance data on the aetiological agents of diarrhoea [8]*.* Furthermore, when an aetiological agent is defined, there is seldom a detailed characterization (source attribution, serotyping, antibiotic susceptibility) of the pathogen. Consequently, we have a limited understanding of how specific enteric pathogens in India relate to the international pool of circulating pathogens and the role of antimicrobial resistance (AMR) in facilitating their success. We previously reported a high prevalence of NTS exhibiting resistance to third-generation cephalosporins in northern India [9]. Here, for what is believed to be the first time, we report the isolation of Cip^R^ *S*. *enterica* Kentucky ST198 associated with human NTS disease in India. Through whole-genome sequencing (WGS), we aimed to further characterize these organisms in the context of those circulating internationally and assess the potential role of domesticated animals as infection reservoirs.

## Methods

We conducted sustained surveillance from April 2014 to September 2017 for NTS organisms associated with human diarrhoeal disease at the Post Graduate Institute of Medical Education and Research (PGIMER, Chandigarh, India) and network laboratories in the Indian states of Punjab, Haryana, Rajasthan, Uttarakhand and Himachal Pradesh. Stool samples from patients with community acquired diarrhoea were collected in a sterile container and transported to PGIMER, Chandigarh, in Cary–Blair transport media for further processing. Concurrently, we conducted cross-sectional animal sampling of meat products and farm animals (sheep, goats, pigs and chickens) in the states of Punjab, Haryana, Himachal Pradesh and Chandigarh from markets and farms in same areas from where human samples were collected. The meat shop and farm owners were approached, and those who agreed to provide samples were included in the study (Table 1). A loopful (10 µl) of human faecal sample was cultured on MacConkey agar and XLT4 agar and incubated overnight at 37 ˚C to isolate NTS. Additionally, faecal (10 g) and meat samples (25 g) collected in sterile containers were transported on ice to the laboratory at PGIMER and were inoculated into 225 ml buffered peptone water before selective enrichment in Rappaport Vassiliadis broth (100 ml) and sub-culturing onto MacConkey and XLT4 agar [10, 11]. Non-lactose fermenting colonies were confirmed as *Salmonella* spp. using a MALDI-TOF bacterial identification system (Bruker). Minimum inhibitory concentrations for colistin and ciprofloxacin were measured using the Vitek AST card N280 on a Vitek 2 system (bioMérieux) and by Etest (bioMérieux). Additionally, we extracted genomic DNA from overnight cultures before preparing genomic libraries using the Illumina TruSeq library preparation kit. WGS was performed using the V3 MiSeq reagent kit on a MiSeq platform (Illumina) to generate 300 bp paired-end reads. Read quality was assessed using fastqc and before manual trimming. *In silico* Multilocus sequence typing (MLST) and AMR genes were identified using srst2. Sequences were mapped to a reference sequence of *S*. *enterica* Kentucky ST198 strain PU131, accession number CP026327, to detect SNPs [12] using the RedDog v1.4 mapping pipeline (https://github.com/katholt/reddog). Additional *S*. *enterica* Kentucky ST198 sequences were accessed on GenBank using the sra toolkit and a maximum-likelihood tree was reconstructed using a bootstrap value of 100 in RAxML. The tree was rooted using a non-Cip^R^ isolate as the outgroup.

**Table 1.** Animal sample (stools/meat) collection and isolation of NTS and *S*. *enterica* Kentucky

State	Location	Approached		Sampled			No. of samples collected			No. of NTS isolated			No. of *S* *. enterica* Kentucky isolated		
		Farms	Shops	Chicken farms/shops	Pig farms/shops	Goat farms/shops*	Chicken stool†/ meat	Pig stool†/meat	Goat stool†/meat	Chicken stool/ meat	Pig stool/ meat	Goat stool/ meat	Chicken stool/ meat	Pig stool/ meat	Goat stool/ meat
Haryana	Barwala‡	5	8	2/6	0§	2/2	9/6	0/0	2/2	0/0	0/0	0/0	0	0	0
Punjab	Patiala	9	12	2/9	2/4	1/5	12/9	7/4	2/5	0/0	0/0	0/0	0	0	0
	Ropar‡	4	22	4/15	3/7	3/15	114/15	10/7	6/18	17/0	0/0	0/0	8/0	0	0
	Mohali/Balongi‡	6	18	4/7	2/3	6/3	56/7	7/3	12/3	5/0	1/0	0/0	4/0	0	0
	Sangrur	3	5	2/4	1/1	0/2	10/4	3/1	0/2	0/0	0/0	0/0	0	0	0
	Samana	5	3	2/3	0/0	1/3	13/3	0/0	2/3	2/1	0/0	0/0	0	0	0
	Anandpur Sahib	4	8	1/2	0/0	2/2	30/2	0/0	2/2	0/0	0/0	0/0	0	0	0
	Kurali‡	9	18	3/11	2/1	5/4	58/11	5/1	9/4	3/0	1/1	0/0	3/0	0	0
Himachal Pradesh	Nahan	7	3	4/4	0/0§	2/4	27/4	0/0	9/6	1/1	0/0	0/0	0	0	0
	Kangra	4	3	2/3	0/0§	2/4	7/3	0/0	5/4	0/0	0/0	0/0	0	0	0
	Hamirpur	5	2	1/3	0/0§	3/6	4/3	0/0	3/7	0/0	0/0	0/1	0	0	0
Chandigarh	Slaughterhouse||			0¶	Sampled once per week for 30 weeks from March 2014 to October 2014		0/0	25/135	38/135	0/0	20/29	7/2	0	0	0
	Sector 21 market#	0	10	0/9	0#	0#	0/10	0/0	0/0	0/0	0/0	0/0	0	0	0
Total		61	112	27/75	8/16	27/50	340/77	57/151	90/191	28/2	22/30	7/3	15/0	0	0/2

*Goat farms in these areas usually have 5 to 15 animals, from which we sampled 1–3 animals. For goat meat, 250 g meat was bought from 1 to 2 animals from each shop depending on the availability of the animals.

†Freshly passed stool samples were collected at all places except for the slaughterhouse, where we had access to intestinal contents.

‡Poultry farms in these regions are organized big commercial farms with a capacity of 10000–15000 birds, where we sampled 25–30 birds from each of the farms. Other farms sampled were moderate sized with a capacity of 2000–3000 birds, from these 4–5 birds were sampled from each farm. For chicken meat, one bird per vendor was bought from the meat shops in the same area.

§No pig farms were located in this region. Pig rearing is a budding industry in North India and pig meat is consumed by a small population. Farm size usually varies between 5 and 15 animals and most of the pigs reared in this region are transported to north-eastern states of India where pork is vastly consumed. We collected stool samples from 2 to 4 animals per farm.

||The slaughterhouse in Chandigarh functions under the jurisdiction of the Municipal Corporation Chandigarh. It is a mechanized abattoir where goats and pigs are slaughtered, the meat is checked by a food inspector and then transported to the shops in a controlled-temperature transport system. This mechanical abattoir serves the regions of Chandigarh, Punjab and Haryana, and up to 200–250 animals are slaughtered in a day.

¶Chicken is not slaughtered at the slaughterhouse in Chandigarh.

#The sector 21 market is a poultry and fish market, meat from other animals is not available here.

## Results and Discussion

In this cross-sectional surveillance study conducted in multiple sites in northern India, we processed 1912 human diarrhoea stool samples and 906 animal samples (419 meat and 487 stool samples, Table 1). A total of 117 NTS were isolated from humans (*n*=25) and food animals (*n*=92) of which *S*. *enterica* Kentucky was found to be the most commonly isolated serovar, comprising 23/117 (19.7 %) of all NTS isolates. These 23 *S. enterica* Kentucky originated from humans (*n*=6), chickens (*n*=15) and goats (*n*=2). Table S2(a, b) shows the demographic details of the patients from whom NTS and *S*. *enterica* Kentucky were isolated, respectively. Notably, the 15 organisms from poultry were isolated from only three farms all located in the state of Punjab. All (23/23; 100 %) *S*. *enterica* Kentucky were found to be Cip^R^, but susceptible to third-generation cephalosporins (ceftazidime, cefepime), carbapenems (meropenem, imipenem), azithromycin and colistin.

Most human and non-human Indian Cip^R^ ST198 *S*. *enterica* Kentucky (91 %, 21/23) contained genes conferring resistance against gentamicin (*aac3-Id*), β-lactams (*blaTEM1*), sulphonamides (*sulI*) and tetracycline (*tetA, tetR*). In the majority of isolates, these AMR genes were located within a 43 kb *Salmonella* genomic island (SGI1), integrated into the chromosome between *trmE/yidY* genes. This genomic island showed 88–89 % identity with SGI-1K; a variant of SGI1 described previously in *S*. *enterica* Kentucky [13]. Resistance to streptomycin is used as an epidemiological marker for identification of the characteristic ACSSuT (ampicillin, chloramphenicol, streptomycin, sulphonamides and tetracyclines) phenotype of resistance in *S. enterica* serovar Typhimurium DT104. Streptomycin resistance is mostly due to aminoglycoside-modifying phosphotransferases, which are encoded by *strA* [*aph(3′)-ib*] and *strB* [*aph (6′)-id*] genes present on the transposon *tnpA1133*. These *strA/B* genes, though absent in our isolates, were present in 14/126 (11.11 %) of the GenBank sequences accessed in this study [14].

**Fig. 1.**
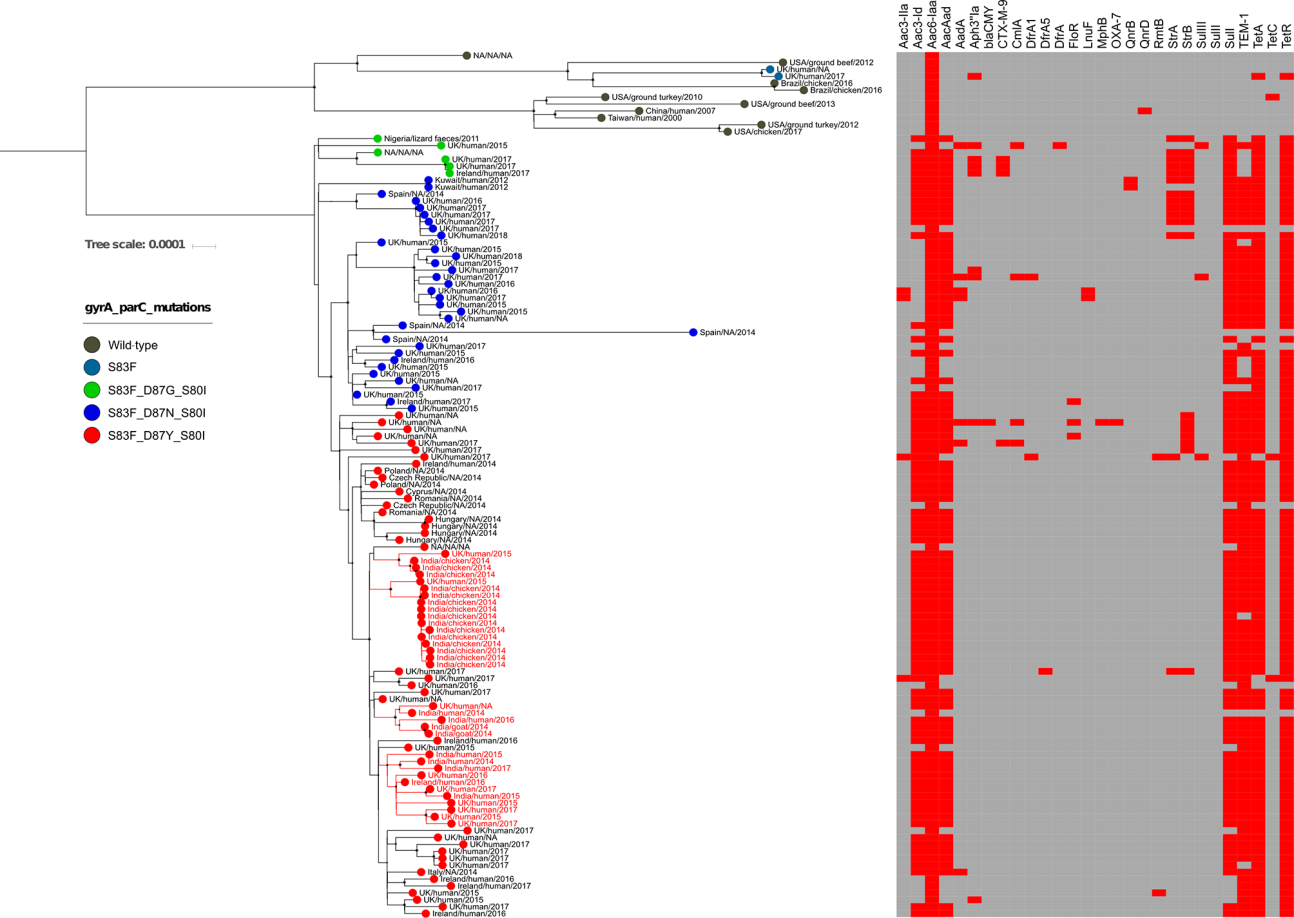
The phylogenetic structure of *S.enterica* Kentucky from WGS. The maximum-likelihood phylogenetic tree was reconstructed from SNPs across the *S*. *enterica* Kentucky genome. The country, host species and year of isolation are identified on the branch tips; Indian isolates from this study are in red script. The terminal circles are coloured according to the mutations in *gyrA* and *parC* genes (see key). The heat map corresponds to the presence (red) and absence (grey) of AMR genes.

WGS permitted assessment of the population structure and investigation of how the organisms from northern India were related to other global *S*. *enterica* Kentucky isolates (Fig. 1). All contemporary *S*. *enterica* Kentucky from India belonged to ST198 and were closely related to previously described Cip^R^ *S*. *enterica* Kentucky isolated outside India. The Cip^R^ *S*. *enterica* Kentucky ST198 isolates formed a single major clade, which had low genetic diversity and was distantly related to the Cip^S^ ST198, suggesting all global Cip^R^ organisms are a newly emerging clone originating from a single ancestor [5].

As reported previously [7], among the three different mutation profiles (S83F_D87G_S80I, S83F_D87N_S80I and S83F_D87Y_S80I) we also found S83F_D87Y_S80I mutations to be associated with Cip^R^ across the tree. Each of the three groups of Cip^R^ ST198 formed an independent tight cluster (with high supported bootstrap values) suggesting that each Cip^R^ ST198 sub-clone has emerged once and then expanded internationally. All human and non-human Cip^R^ ST198 isolates from India had an identical Cip^R^ mutation profile (S83F_D87Y_S80I), indicative of the emergence of a single Cip^R^ organism followed by local establishment. Our data suggest that Cip^R^ *S*. *enterica* Kentucky ST198 may have originated in India, where it expanded throughout poultry and other livestock and occasionally causes infections in humans. The earliest documented multi drug resistant (MDR) *S*. *enterica* Kentucky isolate, which was resistant to nalidixic acid and ciprofloxacin (the strain had a minimum inhibitory concentration of 2 μg ml^−1^, which would be deemed ciprofloxacin resistant by the current breakpoint of 1 μg ml^−1^), was reported from a spice imported into Australia from India [15]. The Cip^R^ ST198 isolates from chickens and goats fell into separate clusters, suggesting that they circulate independently from each other. However, we identified several cases where Cip^R^ ST198 from chickens and goats clustered alongside human isolates, indicative of possible transmission between animals and humans, potentially via the consumption of contaminated foods/contact with animals. Additionally, there were some examples where human and non-human Cip^R^ ST198 isolates from India clustered together with isolates from the UK, suggesting an epidemiological link to international travellers between India and UK.

## Conclusions

This is believed to be the first study originating from South Asia reporting the presence of the epidemic Cip^R^ *S*. *enterica* Kentucky clone ST198 in humans and animals. We conclude that Cip^R^ *S*. *enterica* Kentucky is endemic in humans in India, and likely associated with animal reservoirs including chickens and goats. We speculate that *S*. *enterica* Kentucky infection has been endemic in humans and in India for some time, but has not been reported due to the lack of sustained surveillance. This study highlights the role that animals and humans in India play in the circulation of emerging AMR enteric pathogens, and shows how WGS data is vital for integrating international surveillance systems.

## Data bibliography

Shah DH, Paul NC, Guard J. *S.enterica* serovar Kentucky ST198 strain PU131, accession number CP026327 (2018).

## Supplementary Data

Supplementary File 1

Supplementary File 2
