# Supplementary material for: Endemic fluoroquinolone-resistant Salmonella enterica serovar Kentucky ST198 in northern India
Source: Microb Genom. 2019 Jun 5;5(7):e000275. doi: 10.1099/mgen.0.000275 (PMC6700665; doi:10.1099/mgen.0.000275)
Supplement: Supplementary File 2 [file mgen-5-275-s002.pdf]

Table 2a: Demographic details of NTS isolates.

S. no	Location	No of samples collected	Age groups	Male	Female	No of NTS isolated	No of *S.* Kentucky isolated
1.	Chandigarh	648	0-2	2	0	2	1
			>2-5	0	0	0	
			>5-15	0	0	0	
			>15-40	2	3	5	
			>40	1	2	3	
		Sub-total		5	5	10	
2.	Haryana	340	0-2	3	2	5	1
			>2-5	1	0	1	
			>5-15	0	0	0	
			>15-40	0	0	0	
			>40	0	1	1	
		Sub-total		4	3	7	
3.	Himachal Pradesh	262	0-2	0	0	0	0
			>2-5	0	0	0	
			>5-15	0	0	0	
			>15-40	0	0	0	
			>40	0	1	1	
		Sub-total		0	1	1	
4.	Uttarakhand	189	0-2	1	0	1	0
			>2-5	0	0	0	
			>5-15	0	0	0	
			>15-40	0	0	0	
			>40	0	0	0	
		Sub-total		1	0	1	
5.	Rajasthan	87	0-2	0	0	0	0
			>2-5	0	0	0	
			>5-15	0	0	0	
			>15-40	0	0	0	
			>40	0	0	0	
		Sub-total		0	0	0	
6.	Punjab	386	0-2	0	0	0	4
			>2-5	1	0	1	
			>5-15	1	0	1	
			>15-40	0	1	1	
			>40	1	2	3	
		Sub-total		3	3	6	
	Total	1912		13	12	25	6

Table 2b.Age, sex, region and clinical presentation of patients from whom *Salmonella* Kentucky was isolated.

S. no	Sample id	Source	Year of isolation	Location	Patient details	Diagnosis
1.	768/14	Human	2014	Amritsar, Punjab	60/F	Acute myeloid leukaemia with acute gastroenteritis
2.	H243	Human	2014	Nayagaon, Punjab	66/F	Acute gastroenteritis
3.	1185/15	Human	2015	Mohali, Punjab	50/M	Acute gastroenteritis with Chronic kidney disease
4.	S207	Human	2015	Nawanshahr, Punjab	23/F	Acute gastroenteritis with Splenomegaly
5.	1414/16	Human	2016	Chandigarh	8 months/M	Chronic diarrhoea
6.	M706	Human	2017	Panchkula, Haryana	2/M	Acute gastroenteritis
